# The 2-Year Leakage Index and Quantitative Microaneurysm Results of the RECOVERY Study: Quantitative Ultra-Widefield Findings in Proliferative Diabetic Retinopathy Treated with Intravitreal Aflibercept

**DOI:** 10.3390/jpm11111126

**Published:** 2021-11-01

**Authors:** Amy S. Babiuch, Charles C. Wykoff, Sari Yordi, Hannah Yu, Sunil K. Srivastava, Ming Hu, Thuy K. Le, Leina Lunasco, Jamie Reese, Muneeswar G. Nittala, SriniVas R. Sadda, Justis P. Ehlers

**Affiliations:** 1 Cole Eye Institute, Cleveland Clinic, Cleveland, OH 44106, USA; babiuca@ccf.org (A.S.B.); SRIVASS2@ccf.org (S.K.S.); REESEJ3@ccf.org (J.R.); 2 The Tony and Leona Campane Center for Excellence for Image-Guided Surgery and Advanced Imaging Research, Cole Eye Institute, Cleveland Clinic, Cleveland, OH 44106, USA; yordis2@ccf.org (S.Y.); hum@ccf.org (M.H.); thuyle1561@gmail.com (T.K.L.); LUNASCL@ccf.org (L.L.); 3 Retina Consultants of Texas, Kingwood, TX 77339, USA; ccwmd@retinaconsultantstexas.com (C.C.W.); hannah.yu@houstonretina.com (H.Y.); 4 Blanton Eye Institute, Houston Methodist Hospital, Houston, TX 77030, USA; 5 Department of Quantitative Health Sciences, Lerner Research Institute, Cleveland Clinic Foundation, Cleveland, OH 44195, USA; 6 Doheny Eye Institute, Los Angeles, CA 90033, USA; mnittala@doheny.org (M.G.N.); ssadda@doheny.org (S.R.S.)

**Keywords:** anti-vascular endothelial growth factor, diabetic macular edema, diabetic retinopathy, leakage index, microaneurysms, intravitreal aflibercept, neovascularization, optical coherence tomography, ultra-widefield fluorescein angiography

## Abstract

Eyes with proliferative diabetic retinopathy (PDR) have been shown to improve in the leakage index and microaneurysm (MA) count after intravitreal aflibercept (IAI) treatment. The authors investigated these changes via automatic segmentation on ultra-widefield fluorescein angiography (UWFA). Forty subjects with PDR were randomized to receive either 2 mg IAI every 4 weeks (Arm 1) or every 12 weeks (Arm 2) through Year 1. After Year 1, Arm 1 switched to quarterly IAI and Arm 2 to monthly IAI through Year 2. By Year 2, the Arm 1 leakage index decreased by 43% from Baseline (*p* = 0.03) but increased by 59% from Year 1 (*p* = 0.04). Arm 2 decreased by 61% from Baseline (*p* = 0.008) and by 31% from Year 1 (*p* = 0.12). Both cohorts exhibited a significant decline in MAs from Baseline to Year 2 (871 to 410; *p* < 0.001; 776 to 207; *p* < 0.001, respectively). Subjects with an improved leakage and MA count showed a more significant improvement in the Diabetic Retinopathy Severity Scale (DRSS) score. Moreover, central subfield thickness (CST) was positively associated with changes in the leakage index. In conclusion, the leakage index and MA counts significantly improved from Baseline following IAI treatment, and monthly injections provided a more rapid and sustained reduction in these parameters compared with quarterly injections.

## 1. Introduction

Currently, over 34.2 million people in the United States have Type I or Type II diabetes mellitus, and 88 million US adults have prediabetes [1]. Diabetic retinopathy (DR), the most common form of diabetic-related eye disease, is the leading cause of visual impairment and blindness in working-age Americans, and is expected nearly to double from 2010 to 2050 (7.7 million to 14.6 million) [2]. Approximately one-third of patients with DR are estimated to have vision-threatening complications such as proliferative diabetic retinopathy (PDR) or diabetic macular edema (DME) [3].

Vision-threatening complications in DR arise from prolonged hyperglycemia causing a cascade of biochemical pathways. These pathways lead to oxidative stress in the retinal vasculature and subsequently cause microvascular dysfunction. Ultimately, the development of retinal ischemia in DR triggers the release of the Vascular Endothelial Growth Factor (VEGF), a signaling protein that potentiates this cycle, leading to progressive ischemia and resultant neovascularization, the hallmark of proliferative diabetic retinopathy (PDR). The use of fluorescein angiography (FA) can demonstrate microaneurysms (MAs), vascular leakage, retinal ischemia, and neovascularization (NV) [3]. Retinal vision threatening complications of PDR can include traction retinal detachment, vitreous hemorrhage, macular edema, and ischemic changes [4,5,6]. If neovascularization occurs in the anterior chamber, neovascular glaucoma may develop with potential complications such as hyphema, optic nerve disease, and ensuing vision loss [7].

Classically, treatment for PDR has been panretinal photocoagulation (PRP). Its efficacy in halting disease progression was demonstrated in multiple studies over the last 50 years [8,9,10,11]. Therapies specifically targeting the neovascularization process, in particular the VEGF blockade, recently showed the potential to preserve and possibly reverse underlying retinal damage and vision loss that can occur in the setting of PDR. The Diabetic Retinopathy Clinical Research Network (DRCR.net) Protocol S study demonstrated that intravitreal anti-VEGF therapy was non-inferior to PRP and was potentially associated with fewer complications [12]. Unlike PRP, which slows disease progression, anti-VEGF treatment can not only slow disease progression but can also improve the Diabetic Retinopathy Severity Scale (DRSS) score, as defined by the Early Treatment Diabetic Retinopathy Study (ETDRS). Several longitudinal studies demonstrated at least a 2-step improvement in DRSS with anti-VEGF therapy compared to sham or PRP treatment over 2, 3, and 5 years [13,14,15,16], with some showing improved visual acuity, slower progression, and decreased macular edema when compared to PRP [14,15].

The ETDRS research group previously defined DRSS using 7-frame fundus photographs to characterize and monitor changes in the retina over time [15,17,18]. More recently, ultra-widefield photography and ultra-widefield fluorescein angiography (UWFA) have allowed for a wider field of view of up to 200°, making it possible to monitor panretinal changes with a single image. In order to standardize and more effectively track these changes, automated programs were employed to measure angiographic parameters such as microaneurysm count, leakage, and ischemia [19,20,21,22,23,24,25]. Although the effects of anti-VEGF on PDR features are recognized, there is limited information regarding the effect on quantitative angiographic features on UWFA. The purpose of this study is to assess longitudinally the MA count and leakage occurring on UWFA images over a 2-year period in patients with PDR being treated with a fixed-interval intravitreal aflibercept.

## 2. Materials and Methods

RECOVERY (Intravitreal Aflibercept for Retinal Non-Perfusion in Proliferative Diabetic Retinopathy) is a prospective, randomized, multicenter, and open-label clinical trial (NCT02863354; IND131056; https://clinicaltrials.gov/ct2/show/NCT02863354, accessed on 8 September 2021) as previously described [26,27,28]. Institutional Review Board (IRB)/Ethics Committee approval was obtained (Sterling IRB); the tenets of the Declaration of Helsinki were followed, and the study is in accord with the Health Insurance Portability and Accountability Act of 1996. All subjects were enrolled at the Retina Consultants of Texas (Houston, Katy, and Woodlands, TX, USA).

### 2.1. Inclusion and Exclusion Criteria

Inclusion in the study required PDR, an EDTRS best corrected visual acuity of ≥19, and substantial nonperfusion (≥20 disk areas). Subjects with previous anti-VEGF treatment, a history of PRP or vitreoretinal surgery, a clinically relevant DME, or ≥320 μm central retinal thickness in the study eye were excluded.

### 2.2. Study Design

At enrollment, subjects were consented for participation in the trial and then randomized 1:1. Arm 1 (*n* = 20) received 2 mg (0.05 mL) intravitreal aflibercept injection (IAI) every month (q4weeks; q28 ± 7 days). Arm 2 (*n* = 20) received 2 mg IAI quarterly (q12weeks; q3 months). After Year 1, treatment regimens were crossed over, and the Arm 1 began receiving quarterly injections whereas the Arm 2 began receiving monthly injections until the study end at Year 2. Each visit consisted of BCVA testing with the ETDRS chart, a slit lamp examination, an indirect ophthalmoscope examination, and spectral-domain optical coherence tomography (SD-OCT) scanning.

If a patient undergoing quarterly treatment met prespecified criteria at any visit, they were to be treated every 4 weeks with IAI. These criteria were (1) increased neovascularization, (2) BCVA decrease by ≥5 letters due to progressive DME or PDR, (3) worsening of DME causing vision loss, (4) total area of retinal ischemia increasing by 10%. If a patient was then determined to be either stable or improved by these criteria, they were continued on their pre-specified treatment. All subjects could also receive rescue treatment with PRP if PDR progressed despite IAI treatment.

UWFA images were obtained after dilation at Baseline, Year 1, and Year 2 via the Optos 200Tx (Optos plc, Dunfermline, UK). Images were taken during early (~0–60 s after fluorescein dye injection), middle (~60–180 s), and late (~300 s) phases of the angiogram. The images were then transformed to stereographic projection images using proprietary software available from the manufacturer based on ray tracing each pixel with a combined optical model of the imaging device, as previously described [15].

### 2.3. Outcome Measures

Early and late images were analyzed by an automated assessment platform to provide quantitative image feature extraction, including the MA count and leakage index [21,24,25]. Two masked readers reviewed the program’s automated segmentation and manually corrected any errors. Three pre-specified macula-centered zones were also created to enable the calculation of zonal changes. Zone 1 defines a posterior zone with a 3-disc-diameter boundary including the fovea. Zone 2 defines a midperiphery zone with a 6-disc-diameter boundary centered at the fovea. Zone 3 defines a far periphery zone with a 9-disc-diameter boundary centered at the fovea.

The MA count analysis was performed in the mid-arteriovenous phase of the fluorescein angiogram. MAs were defined as small dots which were significantly hyperfluorescent compared to the surrounding choroidal fluorescence in early-mid-phase fluorescein angiography images [21,28]. For leakage analysis, an early and late phase UWFA image was selected. Leakage was defined as an area of increased hyperfluorescence in the late phase compared to the early phase. The panretinal leakage index was calculated as the area of leakage divided by the total analyzable retinal area. Values were multiplied by 100 to express as a percentage [27,28]. DRSS scores were derived in accordance with the Early Treatment Diabetic Retinopathy Study (ETDRS).

### 2.4. Statistical Analysis

Statistical analysis was performed with R software version 3.4.3 (www.r-project.org; last accessed on 8 September 2021) and the Microsoft Excel statistical function to compare mean differences in each group between Baseline and Year 1, Baseline and Year 2, and between Year 1 and Year 2. Differences in groups were also determined per the region of interest, including the total analyzable retina, Zone 1, Zone 2, and Zone 3. RECOVERY data were also analyzed by visit: Baseline, Year 1, and Year 2.

## 3. Results

### 3.1. Clinical Characteristics

Forty subjects were randomly allocated 1:1 to Arm 1 (*n* = 20) and Arm 2 (*n* = 20). All characteristics were similar between Arm 1 and Arm 2 at Baseline. In Arm 1, the mean age was 47.7 ± 12.1 years, with 9 (45%) male and 11 (55%) female subjects. Four (20%) subjects had Type I Diabetes Mellitus (DM) while 16 (80%) were diagnosed with Type II DM, with an average Baseline glycated hemoglobin A1C (HbA1c) of 9.7 ± 2.2%. Baseline BCVA was 20/32 (ETDRS 77.8 ± 6.6), and the central subfield thickness (CST) was 279.7 ± 38.8 μm. In Arm 2, the mean age was 48.3 ± 12.0 years. Furthermore, 12 (60%) were male and 8 (40%) were female; 5 (25%) subjects had Type I DM while 15 (75%) had Type II DM. Mean Baseline HbA1c was 9.2 ± 2.7%. At Baseline, mean BCVA was 20/25 (ETDRS 79.0 ± 8.2), and the mean CST was 276.4 ± 22.7 μm.

All subjects received an average of 14.4 ± 3.8 total injections up to Year 2, with no overall significant difference between the cohorts (*p* = 0.16). Arm 1 received a mean 10.95 ± 1.8 injections between Baseline and Year 1, and 4.25 ± 1.5 injections between Year 1 and Year 2. Arm 2 received an average of 3.9 ± 0.5 injections by Year 1, and 9.6 ± 4.4 injections between Year 1 and Year 2. All Baseline characteristics are listed in Table 1.

### 3.2. Panretinal Leakage Index

**Figure 1:**
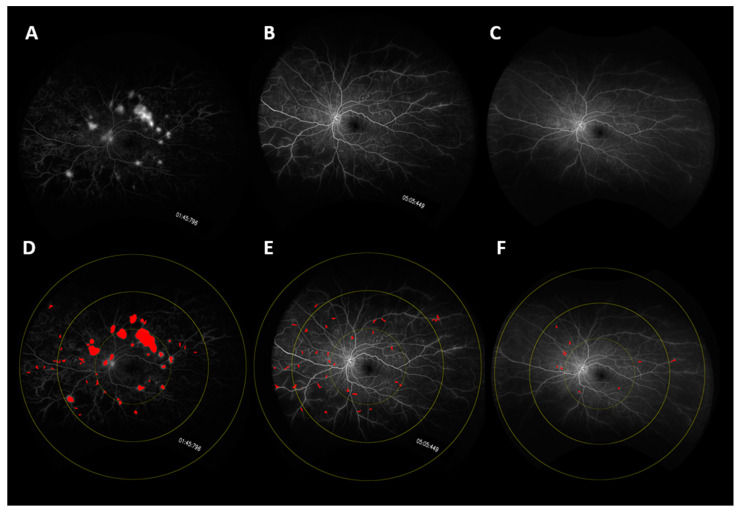
UWFA fundus images of a representative case showing changes in leakage over time. Fundus images are shown at (**A**) Baseline, (**B**) Year 1, (**C**) Year 3. Leakage mask over-lays are shown at (**D**) Baseline, (**E**) Year 1, and (**F**) Year 2. (**D**–**F**) also show the macula-centered concentric rings: Zone 1 defines a posterior zone with a 3-disc-diameter boundary including the fovea. Zone 2 defines a mid-periphery zone with a 6-disc-diameter boundary centered at the fovea. Zone 3 defines a far periphery zone with a 9-disc-diameter boundary centered at the fovea.

**Figure 2:**
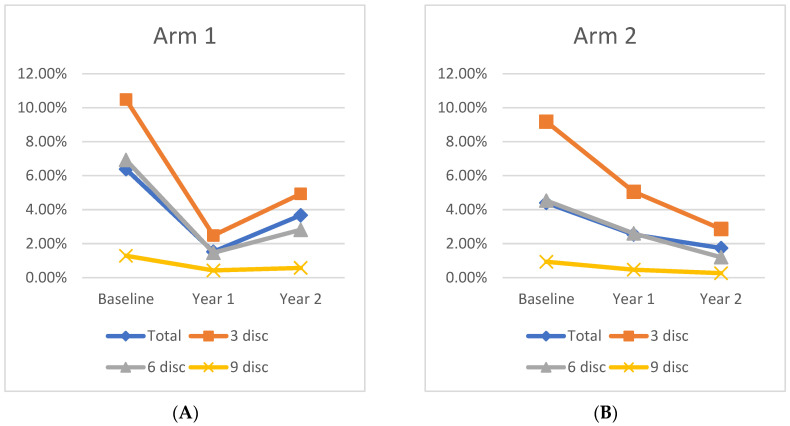
Change in leakage index over time for (**A**) Arm 1 and (**B**) Arm 2 in each region.

At Baseline, the mean panretinal leakage index in Arm 1 and Arm 2 was 6.4 ± 5.4% and 4.4 ± 2.5% (*p* = 0.15), respectively. At Year 1, the total leakage index of Arm 1 improved significantly to 1.5 ± 1.45% (*p* < 0.001). Following crossover to q12week IAI dosing, the Arm 1 leakage index increased to 3.7 ± 3.5% at Year 2, which is a 43% decrease from Baseline (*p* = 0.03), but a 59% increase following the transition to quarterly dosing (*p* = 0.03). In Arm 2, panretinal leakage significantly improved to 2.5 ± 2.7% (*p* = 0.04) at Year 1. Following treatment crossover to q12week IAI, further improvement was demonstrated as 1.7 ± 3.4% (31% decrease; *p* = 0.12) through Year 2, but this was not a significant change from Year 1. However, the improvement from Baseline to Year 2 remained significant, demonstrating a 61% decrease from Baseline (*p* = 0.008). A representative case is shown in Figure 1, and changes in the mean leakage index in both arms are presented graphically in Figure 2.

Overall, 26 subjects, 46% (12/26) from Arm 1 and 54% (14/26) from Arm 2, showed an improvement in the panretinal leakage index from Baseline to Year 2, while 6 subjects, 83% (5/6) from Arm 1 and 17% (1/6) from Arm 2, worsened in the leakage index. Those with the most improved leakage index had a significantly higher leakage index at Baseline (6.08 ± 4.93% vs. 3.60 ± 1.92%; *p* = 0.02) and also demonstrated a greater decrease in CST from Baseline to Year 1 (−29.17 ± 28.2 vs. −24.3 ± 16.0; *p* = 0.009) and to Year 2 (−31.83 ± 32.6 vs. −28.3 ± 16.1; *p* = 0.03). Notably, all subjects in Arm 1 (19/19) showed an improvement in the panretinal leakage index from Baseline to Year 1. However, in Arm 2, only 13/18 (72%) subjects showed an improvement in the leakage index before treatment crossover, but these subjects showed an overall improvement in the panretinal leakage index from Baseline to Year 2 (−4.10 ± 1.64%). The other five subjects in Arm 2 who had a worsening panretinal leakage index before crossover (5/18; 28%) demonstrated an overall increase in the panretinal leakage index from Baseline to Year 2 (1.42 ± 3.50%; *p* = 0.046).

### 3.3. Zonal Leakage Index

**Figure 3:**
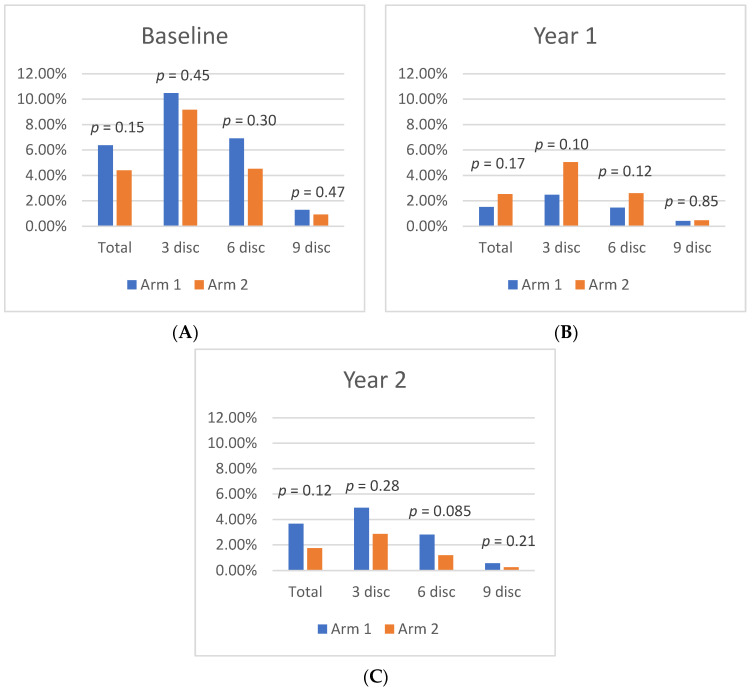
Differences in leakage index per region between Arm 1 and Arm 2 at (**A**) Baseline, (**B**) Year 1, and (**C**) Year 2.

At Baseline, mean leakage index values in Zone 1, Zone 2, and Zone 3 were similar between Arm 1 and Arm 2. By Year 1, both cohorts exhibited a significant leakage index improvement in Zone 1, as Arm 1 decreased from 10.5 ± 6.3% at Baseline to 2.5 ± 2.3% (*p* < 0.01) at Year 1, while Arm 2 decreased from 9.2 ± 4.2% at Baseline to 5.1 ± 5.9% (*p* = 0.02) at Year 1. Moreover, in Arm 1, there was significant leakage index improvement in Zone 2 (6.9% to 1.5%; *p* = 0.03) and Zone 3 (1.3% to 0.4%; *p* = 0.05). Arm 2 had a significant improvement in Zone 2 (4.5% to 2.6%; *p* = 0.045) from Baseline to Year 1, and Zone 3 had a nonsignificant decrease in the leakage index (0.9% to 0.5%; *p* = 0.17). Differences in the leakage index per Zone are illustrated in Figure 3.

Following treatment crossover, the Zone 1 leakage index increased significantly in Arm 1, but continued to decrease in Arm 2. At Year 1, the leakage index of Arm 1 in Zone 1 was 2.5% and increased to 4.9% (*p* = 0.01) by Year 2, representing a 53% decrease from Baseline (*p* = 0.006). In Arm 2, the leakage index of Zone 1 decreased from 5.1% to 2.9% (*p* = 0.05) by Year 2, which represents a 68% decrease from Baseline (*p* = 0.001). This pattern was also observed in Zone 2 and Zone 3 in both cohorts from Year 1 to Year 2, although the only significant change was seen in Zone 2 of Arm 2, which decreased to 1.2% at Year 2, a 54% decrease from Year 1 (*p* = 0.01), and a 73% decrease from Baseline (*p* < 0.001).

### 3.4. Microaneurysms

**Figure 4:**
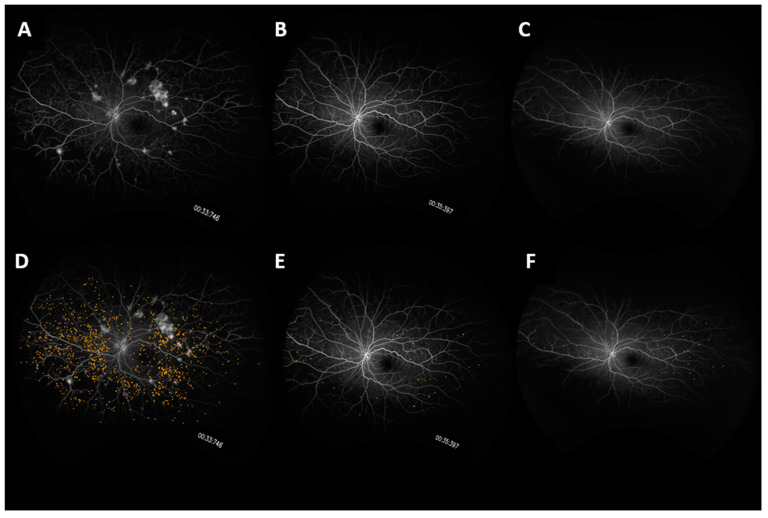
UWFA fundus images of a representative case showing changes in microaneurysm count over time. Fundus images are shown at (**A**) Baseline, (**B**) Year 1, (**C**) Year 3. Microaneurysm mask overlays are shown at (**D**) Baseline, (**E**) Year 1, and (**F**) Year 2.

**Figure 5:**
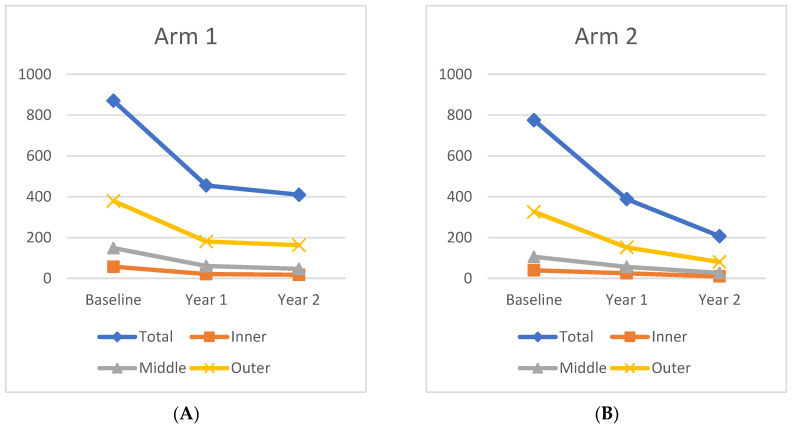
Change in microaneurysm count over time for (**A**) Arm 1 and (**B**) Arm 2 in each region.

From Baseline to Year 1, the panretinal MA count decreased significantly and within each zone. A representative case is shown in Figure 4. In Arm 1, the mean panretinal MA count decreased by 48% (870.7 to 455.4; *p* < 0.001), 63% in Zone 1 (57.5 to 21.2; *p* < 0.001), 62% in Zone 2 (148.8 to 60.6; *p* < 0.001), and 52% in Zone 3 (380 to 181; *p* < 0.001). In Arm 2, the panretinal MA count decreased by 50% (775.7 to 388.6; *p* < 0.001), 38% in Zone 1 (39.6 to 25.1; *p* = 0.009), 47% in Zone 2 (105.8 to 56.4; *p* < 0.001), and 54% in Zone 3 (326.3 to 151.8; *p* < 0.001). However, it is important to note that there was a significant difference in the mean MA count between Arm 1 and Arm 2 at Baseline in Zone 1 (57.5 vs. 39.6 respectively; *p* = 0.03) and in Zone 2 (148.8 vs. 105.8 respectively; *p* = 0.04). The changes in the mean MA count over time can be seen in Figure 5.

**Figure 6:**
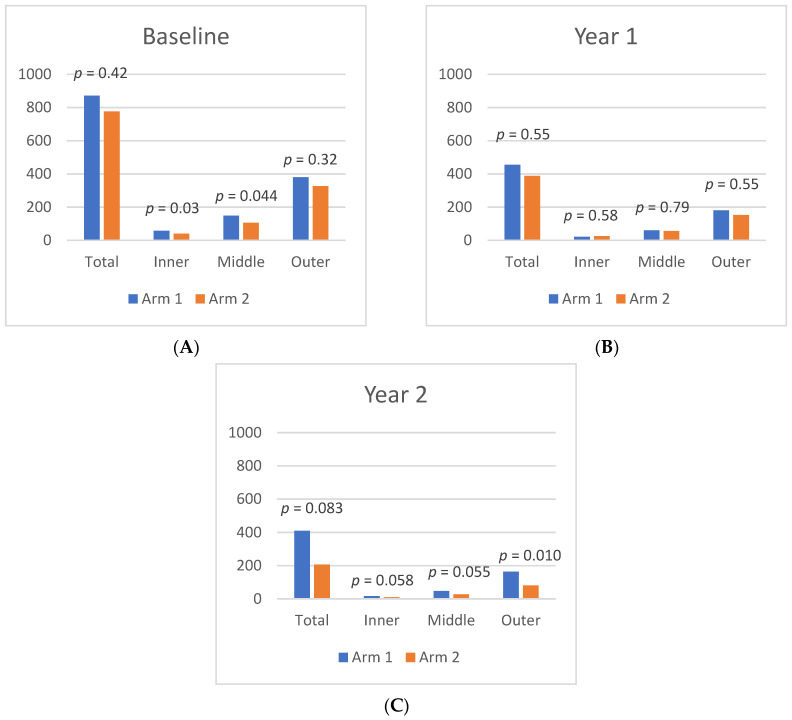
Differences in microaneurysm count per region between Arm 1 and Arm 2 at (**A**) Baseline, (**B**) Year 1, (**C**) and Year 2.

The mean MA count continued to decrease after treatment crossover in both cohorts. When compared to Baseline, the MA count showed significant panretinal improvement and in all zones by Year 2. However, from Year 1 to Year 2, only Arm 2 had significant decreases in the mean MA count in all regions (panretinal, *p* < 0.001; Zone 1, *p* = 0.002; Zone 2, *p* < 0.001; Zone 3, *p* = 0.002). When compared to Baseline, Arm 1 showed a significant panretinal decrease of 53% (870.7 to 409.9; *p* < 0.001), 70% in Zone 1 (57.5 to 17.5; *p* < 0.001), 68% in Zone 2 (148.8 to 46.6; *p* < 0.001), and 57% in Zone 3 (379.6 to 162.9; *p* < 0.001) by Year 2. In Arm 2, the mean panretinal MA burden significantly decreased by 73% (775.7 to 388.6; *p* < 0.001), by 75% in Zone 1 (39.6 to 9.7; *p* < 0.001), by 75% in Zone 2 (105.8 to 26.6; *p* < 0.001), and by 75% in Zone 3 (326.3 to 80.5; *p* < 0.001) from Baseline to Year 2. These region differences are illustrated in Figure 6.

Between Year 1 and 2, eight subjects had both an increase in the MA count and panretinal leakage index, 7/8 (87.5%) of which were from Arm 1 while 1/8 (12.5%) were from Arm 2. The subjects who exhibited a concurrent increased MA count and panretinal leakage index after treatment crossover showed a worsening in DRSS by 1.63 ± 1.68 steps in that time period, whereas those that did not had an improvement of 0.48 ± 1.21 steps (*p* = 0.009). Moreover, between Baseline and Year 2, those with a concurrent worsening MA and panretinal leakage index after crossover had a less significant improvement in DRSS than those that did not (+1.25 ± 1.67 vs. +3.35 ± 1.83; *p* = 0.01).

### 3.5. Functional and Anatomic Outcomes

At Baseline, the mean BCVA of Arm 1 was 20/32 (ETDRS 77.8 ± 6.6). Visual acuity significantly improved in Arm 1 from 20/32 to 20/25 (ETDRS 82.8 ± 7.8; *p* = 0.01) by Year 1, and remained at 20/25 (ETDRS 83.2 ± 9.7) by Year 2. Arm 2 remained at 20/25 between Baseline (ETDRS 79.0 ± 8.2) and Year 1 (ETDRS 80.3 ± 15.6; *p* = 0.69), but improved to 20/20 (ETDRS 87.6 ± 5.8) by Year 2, which is a significant improvement from Baseline (*p* < 0.001). Overall, both cohorts exhibited a significant improvement in visual acuity from Baseline to Year 2 (*p* < 0.001).

**Figure 7:**
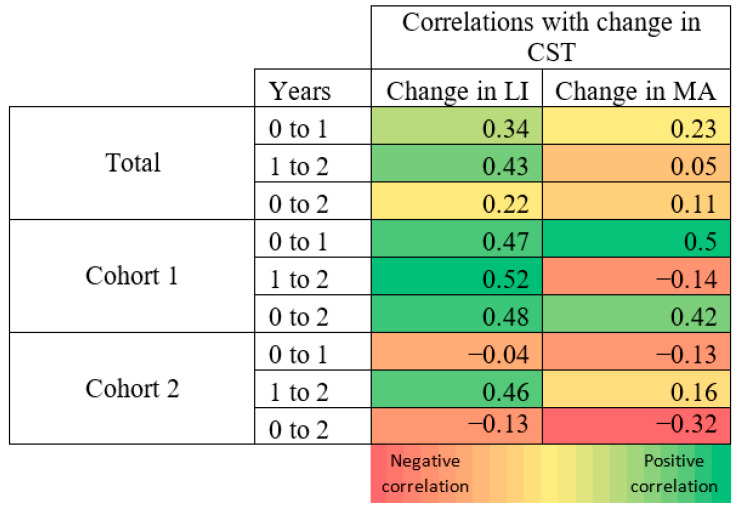
Correlation heat map (red = negative correlation; green = positive correlation). Change in CST correlated to change in leakage index and MA count in the respective time period. Numbers correspond to the Pearson correlation coefficient (r).

The mean central subfield thickness (CST) was similar between Arm 1 and Arm 2 at Baseline—279.7 ± 38.8 μm and 276.4 ± 22.7 μm (*p* = 0.75), respectively. Both groups improved significantly from Baseline to Year 1, decreasing by 11.5% (279.7 to 247.5 μm; *p* < 0.001) in Arm 1 and by 7.6% (276.4 to 255.3 μm; *p* = 0.006) in Arm 2. Arm 1 and Arm 2 also improved significantly from Baseline by Year 2 (246.3 ± 30.9 μm; *p* < 0.001, 249.6 ± 29.2 μm; *p* = 0.01, respectively). The mean change in CST was moderately positively correlated to the change in the leakage index in Arm 1 in all time periods, but was only moderately positively correlated in Arm 2 after switching to monthly dosing at Year 1 (see Figure 7).

DRSS improvement (i.e., decrease in DRSS value) was also apparent in both cohorts. From Baseline to Year 1, 100% (17/17) of subjects in Arm 1 had at least a 1-step improvement, and 70.6% (12/17) had at least a 2-step improvement. In Arm 2, 83.3% (14/16) had at least a 1-step improvement, with 75% (12/16) having improved by at least 2 steps. Between Year 1 and Year 2, 23.5% (4/17) of Arm 1 and 62.5% (10/16) of Arm 2 had at least a 1-step improvement. However, 47.1% (8/17) of subjects in Arm 1 exhibited a worsening of DRSS (i.e., increase in score value) by at least one step during that period compared to 6.25% (1/16) of subjects in Arm 2. Overall, between Baseline and Year 2, Arm 1 had 23.5% (4/17) of subjects maintain the same DRSS, 76.5% (13/17) improved by at least one step, and 58.8% (10/17) improved by at least 2 steps. In that period, 6.3% (1/16) of Arm 2 had stable DRSS, 92.7% (15/16) had at least a 1-step improvement, and 81.3% (13/16) had at least a 2-step improvement.

Subjects with at least a 1-step improvement in DRSS showed a greater reduction in the leakage index over time, as subjects that had an improved DRSS between Baseline and Year 1 showed a reduction of 2.70 ± 4.98% in the leakage index, while those without improvement had a mean increase of 1.38 ± 2.34% (*p* = 0.035) in that period. Similarly, between Year 1 and Year 2, subjects with at least a 1-step DRSS improvement had a mean reduction of the panretinal leakage index of 0.68 ± 1.62%, whereas subjects without improvement in DRSS had a mean increase of 1.86 ± 3.51% (*p* = 0.013) during that period. Although a greater reduction in the MA count over time was also observed in patients with improved DRSS, the change was not significantly different from the group with stable/worsening DRSS from Baseline to Year 1 (−395 vs. −316, respectively; *p* = 0.55), Year 1 to Year 2 (−206 vs. −100; *p* = 0.21), and Baseline to Year 2 (−568 vs. −429; *p* = 0.52).

Nine subjects had worsening DRSS between Year 1 and 2, 8/9 (88.9%) of whom were from Arm 1. When compared to those that did not have worsening DRSS during that period (*n* = 24), there was a significant difference in the mean change in the panretinal leakage index ( +3.87 ± 3.75%, −0.46 ± 1.66%, respectively; *p* = 0.008) and MA count (+4 ± 246, −205 ± 217, respectively; *p* = 0.04) between Year 1 and 2. When comparing the 8 subjects from Arm 1 with worsening DRSS to the other subjects that had stable/improved DRSS (*n* = 9) after treatment crossover, there was a significant difference in the Baseline MA count, as those in Arm 1 with worsening DRSS had a Baseline mean MA count of 695 ± 411 whereas those that did not worsen had 1098 ± 382 (*p* = 0.05).

In Arm 1, among the 9 subjects that had DRSS improvement from Baseline to Year 1, five (55.6%) had stable DRSS from Year 1 to Year 2 whereas 4 (44.4%) continued to improve. The panretinal leakage index at Baseline in the stable group was 4.32 ± 3.12% and was 9.25 ± 1.97% (*p* = 0.02) in the group that showed improvement, and CST measurements were 265.8 ± 36.57 μm and 310.25 ± 18.25 μm (*p* = 0.05), respectively. The change in the panretinal leakage index after crossover was also significantly different in these groups, as the stable group’s leakage index decreased by 1.64 ± 3.97% whereas the improved group’s leakage index decreased by 8.17 ± 1.63% (*p* = 0.02). The change in CST was also greater in the improved group, decreasing by a mean of 65.0 ± 22.73 μm by Year 1 and 71.0 ± 25.14 μm by Year 2, while the stable group decreased by 21.8 ± 16.67 μm (*p* = 0.02) from Baseline to Year 1, and by 25.8 ± 11.39 μm (*p* = 0.03) by Year 2 in the stable group.

### 3.6. Optimal Responders

Optimal responders were defined as subjects who show an improvement in the panretinal leakage index and MA count between Baseline and Year 2. In that group, 13/25 (52%) were male, 12/25 (48%) were current/previous smokers, and 14/25 (56%) had hypertension at Baseline. Overall, both Arm 1 and Arm 2 had a similar number of optimal responders (12/25, 48% vs. 13/25, 52%, respectively), with the majority having a DRSS of a moderate PDR (DRSS = 65). The distributions of DRSS at Baseline were as follows: 6 high-risk PDR (three subjects in Arm 1, three subjects in Arm 2), 16 moderate PDR (eight subjects in Arm 1, eight subjects in Arm 2), and 3 mild PDR (one subject in Arm 1, two subjects in Arm 2).

Optimal responders demonstrated a number of significant differences when compared to other subjects. Optimal responders had a significantly higher leakage index at Baseline compared to those with a worsening leakage index and/or MA count (6.32 ± 4.88% vs. 3.60 ± 1.92%; *p* = 0.05). Between Baseline and Year 1, the decrease in the leakage index was also greater in the optimal responders (−4.63 ± 5.3% vs. −0.12 ± 3.28%; *p* = 0.03). From Year 1 to Year 2, the optimal responders had a slight increase in the average panretinal leakage, but it was significantly smaller than the other subjects (0.14% vs. 3.62%, respectively, *p* = 0.018).

With regards to DRSS, optimal responders improved significantly from Year 1 to Year 2 by a mean 0.42 ± 1.2 steps, while other subjects demonstrated a worsening of 2.17 ± 1.6 steps (*p* = 0.01). Between Baseline and Year 2, optimal responders also had overall a significantly greater improvement in DRSS, improving by a mean 3.17 ± 2.0 steps compared to other subjects, which only improved by 1.0 ± 0.82 (*p* < 0.01). All non-optimal responders either demonstrated a worsened DRSS after treatment crossover (4/6; 66.7%) or remained with a stable DRSS (2/6; 33.3%).

### 3.7. Complications

An analysis of vision-threatening complications in both cohorts revealed that no subjects developed center-involving DME at any point during the study. In addition, no subject required Panretinal Photocoagulation (PRP) over the 2-year time period; however, vitreous hemorrhage (VH) did occur. When excluding the 3 subjects with VH at Baseline (one from Arm 1, two from Arm 2), VH occurred in 7 out of 37 subjects (18.9%), with 5/7 (71.4%) occurring in Arm 1. Subjects who developed VH after Baseline contained the top 20% of the highest values for the Baseline leakage index, with one subject having a Baseline leakage index of 26.08%, the highest of all subjects. These subjects also had a smaller step improvement in DRSS between Baseline and Year 2 compared to those without VH (1 ± 0.89, 3.21 ± 1.93, respectively; *p* < 0.01), as well as a smaller decrease in CST (−20.3 ± 8.69 μm vs. −48.7 ± 58.4 μm; *p* = 0.03). The occurrence of VH did not show a significant difference in the Baseline leakage index or microaneurysm count. No significant difference in age, BMI, Baseline HbA1C, or duration of diabetes was noted between subjects with VH and those without VH.

In the optimal responders, two (2/25, 8%) had VH at Baseline while three developed VH after Baseline (3/25, 12%). All three optimal responders that developed VH after Baseline were from Arm 1, while the two subjects with Baseline VH were from Arm 2. Notably, the optimal responders that developed VH after Baseline were reported to have VH on examination in an average of 10 out of 18.7 (53.5%) visits: 7.3 mean visits between Baseline and Year 1 and 2.7 mean visits between Year 1 and Year 2. Two subjects developed VH after Baseline that were not optimal responders (both in Arm 1), and they had VH reported in a mean 3.5 out of 20 (17.5%) visits; all of these were reported between Year 1 and Year 2.

## 4. Discussion

The semi-automated analysis of ultra-widefield angiography images has enabled an in-depth assessment of angiographic features. Changes in retinal leakage and microaneurysm burden were recently quantitatively analyzed to allow a more detailed understanding of the effects of anti-VEGF treatment on the retina [5,29,30,31]. Recent studies on patients with DR treated by anti-VEGF showed a significant decrease in retinal leakage and microaneurysm count on UWFA [32,33,34,35,36]. The 1-year RECOVERY results also highlighted the effects of dosing frequency on the leakage index and microaneurysm count dynamics over 52 weeks [27,28].

In this 2-year trial, subjects undergoing IAI therapy for DME exhibited a significant decrease in the leakage index and microaneurysm count from Baseline up to Year 2. However, each cohort had significant differences in the rate of improvement, which is further highlighted after treatment crossover at Year 1. Between Baseline and Year 1, Arm 1 (monthly IAI from Baseline to Year 1) showed significant improvement in panretinal and zonal leakage, while the Arm 2 (q12 week IAI from Baseline to Year 1) cohort had significant improvement in all regions except in Zone 3 (9-disc-diameter). More importantly, after treatment crossover, Arm 1, which switched to q12 week IAI injections, saw a worsening in the panretinal leakage index, increasing significantly from Year 1 to Year 2. In contrast, Arm 2, which switched to q4 week IAI, continued to show improvements in the leakage index in all zones, although those changes were not statistically significant. Microaneurysm burden differences were also apparent between cohorts when comparing changes before and after treatment crossover. From Baseline to Year 1, both cohorts showed significant decreases in the MA count in all zones. However, after crossover, only Arm 2 showed a significant improvement in the mean panretinal MA count and in all zones.

In addition to leakage and MA burden, changes in DRSS scores have been a parameter of significant interest in subjects undergoing anti-VEGF treatment. Specifically, the clinical importance of DRSS changes have been examined in relation to visual acuity outcomes [13,15,37]. A study in 2017 by Ip and colleagues showed that subjects with stable or improved DRSS levels had a greater improvement in BCVA letter scores, whereas those with worsening DRSS had a smaller improvement. Moreover, significant changes in the BCVA letter score (>/=15 letters) were more common in patients with at least a 2-step improvement in DRSS [37]. Another study in 2018 by Dhoot and colleagues demonstrated only Baseline DRSS having a strong association with a 2-step DRSS improvement by Year 2, while age, duration of diabetes, HbA1C, BMI, and BCVA had no correlation [38].

In RECOVERY, most subjects (85%) exhibited an improvement of at least 1 step in DRSS between Baseline and the end of the study at Year 2. Similar to the leakage index and MA count, differences were noted when comparing cohorts before and after treatment crossover. Between Baseline and Year 1, all subjects (100%) in Arm 1 and the majority of Arm 2 (83%) had improved DRSS (decrease in score value). However, after crossover, a substantially higher proportion of Arm 2 subjects continued to improve in DRSS by at least 1 step compared to Arm 1. Moreover, from Baseline to Year 2, a higher ratio of subjects in Arm 2 exhibited a 2-step improvement than subjects in Arm 1. Overall, by the end of the study, a larger proportion of subjects in Arm 2 had an improved DRSS than in Arm 1. Notably, the worsening of DRSS (increase in score value) was only seen after crossover, and the majority of cases were from Arm 1. Overall, the subjects with worsening DRSS after crossover showed a significant worsening in the MA count in that period compared to the significant improvement in the MA count seen in those with stable/improved DRSS. Subjects with worsening DRSS after crossover also showed a significantly larger worsening of the panretinal leakage index during that period. Moreover, there was no significant difference in the age, BMI, Baseline HbA1C, and duration of diabetes noted between subjects in those that improved by 1 step or by 2 steps compared to those who did not improve by Year 2.

Changes in DRSS in relation to the leakage index, MA count, and CST were also investigated. The subjects with an improvement in the DRSS score by at least 1 step showed a greater reduction in the leakage index compared to those without improvement. In Arm 1, subjects with an increased DRSS before treatment crossover and who continued to improve after crossover had a significantly larger leakage index and CST at Baseline compared to those with a stable DRSS after crossover. Moreover, those who continued to improve also had a significantly larger decrease in the leakage index and CST between Baseline and Year 1, and a greater decrease in mean CST was shown from Baseline to Year 2. The change in BCVA was also different between those two groups in Arm 1, as those who continued to improve in DRSS showed a significantly greater increase in ETDRS letters by Year 1. In contrast, subjects in Arm 2 who improved in DRSS before crossover and continued to improve had no significant differences in any parameter compared to subjects who improved before crossover and did not improve after crossover.

The results of this study highlight the potential worsening in the leakage index, MA counts, and DRSS associated with a decreasing IAI frequency from monthly to q12 week dosing. Both the PRIME [39] and PANORAMA [40,41] trials suggested that once an improvement in DRSS is achieved, it can be sustained with q12–16 week intravitreal anti-VEGF, but this was not the case in our study. In contrast, our study showed that a gradual induction of q12 week dosing followed by a more frequent q4 week dosing resulted in better functional and anatomic outcomes overall, especially in terms of leakage and DRSS. An important distinction from both the PRIME study and PANORAMA study is the greater severity of PDR in the current study. This difference in findings supports the need for the frequent monitoring of DR and the potential value of concurrent UWFA imaging. It also suggests that applying a treatment regimen across all patients with PDR may be less efficacious than image-guided personalized therapeutic decision-making.

Additionally, this study and recent publications demonstrate a clear link between the leakage index and DRSS [27,39]. Specifically, the progression of DR and hence the worsening of DRSS may be preceded by increases in the leakage index on UWFA, as evidenced by the PRIME Trial (a prospective, randomized, phase 2 trial that enrolled 40 eyes to assess the safety and efficacy of the real-time leakage index vs. DRSS scoring at monthly visits to guide PRN retreatment in both PDR and NPDR patients) [39]. However, repeated regular UWFAs to assess the leakage index in DR is not without drawbacks, and there is a clear appeal to using DRSS for regular DR monitoring because of its non-invasive nature, efficiency, and ease of image capture.

Traditionally, DRSS has been determined by using a central reading center (CRC). The PANORAMA study demonstrated the necessity for trained readers or CRC for the determination of DRSS, when the disparity between the investigator and CRC reader proved the investigator wrong in 50% of cases [40]. Further, the need for precise DRSS and the ability to monitor changes in DRSS is becoming of greater importance, not just in terms of DR progression, but for morbidity and mortality in DM. A recent study in Diabetes Care by Fahrmann demonstrated for the first time the strong correlation between DCCT-ETDRS levels ≥3 (Level 35; mild NPDR) and cardiovascular complications in young type 1 diabetics [42]. ACCORDION, a 9-year observation follow-up study to ACCORD, emphasizes the benefits of the personalization of DR treatment and the potential harm in treating all patients with a one-size-fits-all approach [43]. The authors concluded that patients should be stratified according to their microvascular complications, as their results revealed that DR patients who have undergone vitrectomy or retinal laser photocoagulation derived benefits from intensive glucose control, while those who had not were at an increased cardiovascular risk. While this study used a binary approach to DR, the opportunity for further research in the relationship between DRSS, cardiovascular disease, and glucose control is an unmet need. Multiple other studies demonstrated the relationship between DR and all-cause mortality, further highlighting the increasing importance of DRSS determination in clinical practice [44,45,46,47].

Specific limitations of this study include the relatively small number of subjects, presence of artifacts such as eyelashes and lenticular opacities, and errors that may occur analyzing different or low-quality images. Strengths of the study include its prospective nature and the standardization of image assessment for each parameter through the use of an image reading center and previously validated techniques in image analysis and quantification.

## 5. Conclusions

The quantitative assessments of UWF imaging in this study and others demonstrate the utility and importance of these algorithms in identifying DRSS and DR progression, but also for the prognostication of glucose control and overall morbidity and mortality in patients with DM [27,28,29,37].

## Figures and Tables

**Table 1:** Baseline Demographics and Ocular Characteristics.

	Arm 1 (*n* = 20)	Arm 2 (*n* = 20)	Total (*n* = 40)	*p*-Value, Arm 1 vs. Arm 2
Age	47.7 ± 12.1	48.3 ± 12.0	48.0 ± 12.1	*p* = 0.88 ^†^
Eye (%)				
Right	10 (50)	10 (50)	20 (50)	*p* = 1.00 *
Left	10 (50)	10 (50)	20 (50)	
Sex (%)				
Male	9 (45)	12 (60)	21 (52.5)	*p* = 0.53 *
Female	11 (55)	8 (40)	19 (47.5)	
Diabetes type (%)				
Type 1	4 (20)	5 (25)	9 (23)	*p* = 1.00 *
Type 2	16 (80)	15 (75)	31 (77)	
Years with Diabetes	16.4 ± 8.9	15.8 ± 9.4	16.1 ± 9.0	*p* = 0.82 ^†^
BMI, kg/m^2^	33.1 ± 6.9	31.8 ± 6.4	32.4 ± 6.6	*p* = 0.54 ^†^
HbA1C, %	9.7 ± 2.2	9.2 ± 2.7	9.4 ± 2.5	*p* = 0.56 ^†^
Lens status (%)				
Phakic	19 (95)	18 (90)	37 (92.5)	*p* = 1.00 *
Pseudophakic	1 (5)	2 (10)	3 (7.5)	
CST, μm	279.7 ± 38.8	276.4 ± 22.7	278 ± 31.8	*p* = 0.75 ^†^
ETDRS, letters	77.8 ± 6.6	79 ± 8.2	78.4 ± 7.5	*p* = 0.64 ^†^
Injections, n	15.2 ± 2.8	13.5 ± 4.5	14.4 ± 3.8	*p* = 0.16 ^†^

* Chi-square test; ^†^ Two-sample *t*-test; BMI = Body Mass Index (kg/m^2^); HbA1c = Glycated hemoglobin A1c (%); CST = Central Subfield Thickness (μm); ETDRS = Early Treatment Diabetic Retinopathy Score.

## Data Availability

All data relevant to the study are included in the article. Additional data may be available by request if for some reason that is required.

## RECOVERY Study Group

Charles C. Wykoff M.D., Ph.D.; Muneeswar G. Nittala M.D.; Brenda Zhou, B.S.; Shaun I.R. Lampen, B.S.; Michael Ip, M.D.; SriniVas Sadda, M.D.; Justis P. Ehlers. M.D.; Sunil K. Srivastava, M.D.; Jamie L. Reese, BSN; Amy Babiuch, M.D.; Katherine Talcott, M.D.; Natalia Figueiredo, M.D.; Jenna Hach, B.A.; Alexander Rusakevich, B.A.; William C. Ou, B.S.; Richard H. Fish; Matthew S. Benz, M.D.; Eric Chen, M.D.; Rosa Y. Kim, M.D.; James C. Major Jr., M.D., Ph.D.; Ronan E. O’Malley, M.D.; David M. Brown, M.D.; Ankoor R. Shah, M.D.; Amy C. Schefler, M.D.; Tien P. Wong, M.D.; Christopher R. Henry, M.D.

## References

[1] Center for Disease Control (2020). National Diabetes Statistics Report.

[2] National Eye Institute (2020). Diabetic Retinopathy Data and Statistics.

[3] Lee R., Wong T.Y., Sabanayagam C. (2015). Epidemiology of diabetic retinopathy, diabetic macular edema and related vision loss. Eye Vis..

[4] Tolentino M.J., Miller J.W., Gragoudas E.S., Jakobiec F.A., Flynn E., Chatzstefanou K., Ferrara N., Adamis A.P. (1996). Intravitreous Injections of Vascular Endothelial Growth Factor Produce Retinal Ischemia and Microangiopathy in an Adult Primate. Ophthalmology.

[5] Campochiaro P.A., Wykoff C.C., Shapiro H., Rubio R.G., Ehrlich J.S. (2014). Neutralization of Vascular Endothelial Growth Factor Slows Progression of Retinal Nonperfusion in Patients with Diabetic Macular Edema. Ophthalmology.

[6] Aiello L.P., Avery R.L., Arrigg P.G., Keyt B.A., Jampel H.D., Shah S.T., Pasquale L.R., Thieme H., Iwamoto M.A., Park J.E. (1994). Vascular Endothelial Growth Factor in Ocular Fluid of Patients with Diabetic Retinopathy and Other Retinal Disorders. N. Engl. J. Med..

[7] Fong D.S., Ferris F.L., Davis M.D., Chew E.Y. (1999). Causes of severe visual loss in the early treatment diabetic retinopathy study: ETDRS report no. 24. Am. J. Ophthalmol..

[8] The Diabetic Retinopathy Study Research Group (1976). Preliminary Report on Effects of Photocoagulation Therapy. Am. J. Ophthalmol..

[9] Early Treatment Diabetic Retinopathy Study Research Group (1991). Early Photocoagulation for Diabetic Retinopathy: ETDRS Report Number 9. Ophthalmology.

[10] Ferris F. (1996). Early photocoagulation in patients with either type I or type II diabetes. Trans. Am. Ophthalmol. Soc..

[11] Riaskoff S. (1981). Photocoagulation Treatment of Proliferative Diabetic Retinopathy. Ophthalmology.

[12] Gross J.G., Glassman A.R., Jampol L.M., Inusah S., Aiello L.P., Antoszyk A.N., Baker C.W., Berger B.B., Bressler N.M., Browning D. (2015). Panretinal Photocoagulation vs Intravitreous Ranibizumab for Proliferative Diabetic Retinopathy. JAMA.

[13] Ip M.S., Domalpally A., Hopkins J.J., Wong P., Ehrlich J.S. (2012). Long-term Effects of Ranibizumab on Diabetic Retinopathy Severity and Progression. Arch. Ophthalmol..

[14] Nguyen Q.D., Brown D.M., Marcus D.M., Boyer D.S., Patel S., Feiner L., Gibson A., Sy J., Rundle A.C., Hopkins J.J. (2012). Ranibizumab for Diabetic Macular Edema. Ophthalmology.

[15] Brown D.M., Schmidt-Erfurth U., Do D.V., Holz F.G., Boyer D.S., Midena E., Heier J.S., Terasaki H., Kaiser P., Marcus D.M. (2015). Intravitreal Aflibercept for Diabetic Macular Edema. Ophthalmology.

[16] Wykoff C.C., Eichenbaum D.A., Roth D.B., Hill L., Fung A.E., Haskova Z. (2018). Ranibizumab Induces Regression of Diabetic Retinopathy in Most Patients at High Risk of Progression to Proliferative Diabetic Retinopathy. Ophthalmol. Retin..

[17] Ip M.S., Domalpally A., Sun J., Ehrlich J.S. (2015). Long-term Effects of Therapy with Ranibizumab on Diabetic Retinopathy Severity and Baseline Risk Factors for Worsening Retinopathy. Ophthalmology.

[18] Early Treatment Diabetic Retinopathy Study Research Group (1991). Grading Diabetic Retinopathy from Stereoscopic Color Fundus Photographs—An Extension of the Modified Airlie House Classification: ETDRS Report Number 10. Ophthalmology.

[19] Ehlers J.P., Jiang A.C., Boss J.D., Hu M., Figueiredo N., Babiuch A., Talcott K., Sharma S., Hach J., Le T.K. (2019). Quantitative Ultra-Widefield Angiography and Diabetic Retinopathy Severity: An Assessment of Panretinal Leakage Index, Ischemic Index and Microaneurysm Count. Ophthalmology.

[20] Jiang A., Srivastava S., Figueiredo N., Babiuch A., Hu M., Reese J., Ehlers J.P. (2019). Repeatability of automated leakage quantification and microaneurysm identification utilising an analysis platform for ultra-widefield fluorescein angiography. Br. J. Ophthalmol..

[21] Ehlers J.P., Wang K., Vasanji A., Hu M., Srivastava S.K. (2017). Automated quantitative characterisation of retinal vascular leakage and microaneurysms in ultra-widefield fluorescein angiography. Br. J. Ophthalmol..

[22] Rabbani H., Allingham M.J., Mettu P.S., Cousins S.W., Farsiu S. (2015). Fully Automatic Segmentation of Fluorescein Leakage in Subjects with Diabetic Macular Edema. Investig. Opthalmology Vis. Sci..

[23] Zhao Y., MacCormick I.J.C., Parry D.G., Leach S., Beare N., Harding S.P., Zheng Y. (2015). Automated Detection of Leakage in Fluorescein Angiography Images with Application to Malarial Retinopathy. Sci. Rep..

[24] Tanchon C., Srivastava S.K., Ehlers J.P. (2015). Automated Quantitative Analysis of Leakage and Ischemia for Ultra-widefield Angi-ography in Retinal Vascular Disease. Investig. Ophthalmol. Vis. Sci..

[25] Tan C.S., Chew M.C., van Hemert J., Singer M., Bell D., Sadda S.R. (2015). Measuring the precise area of peripheral retinal non-perfusion using ultra-widefield imaging and its correlation with the ischaemic index. Br. J. Ophthalmol..

[26] Wykoff C.C., Nittala M.G., Zhou B., Fan W., Velaga S.B., Lampen S.I., Rusakevich A., Ehlers J.P., Babiuch A., Brown D.M. (2019). Intravitreal Aflibercept for Retinal Nonperfusion in Proliferative Diabetic Retinopathy: Outcomes from the Randomized RECOVERY Trial. Ophthalmol. Retin..

[27] Babiuch A.S., Wykoff C.C., Srivastava S.K., Talcott K., Zhou B., Hach J., Hu M., Reese J.L., Ehlers J.P. (2020). Retinal leakage index dynamics on ultra-widefield fluorescein angiography in eyes treated with intravitreal aflibercept for proliferative diabetic retinopathy in the recovery study. Retina.

[28] Babiuch A., Wykoff C.C., Hach J., Srivastava S., Talcott K.E., Yu H.J., Nittala M., Sadda S., Ip M.S., Le T. (2020). Longitudinal panretinal microaneurysm dynamics on ultra-widefield fluorescein angiography in eyes treated with intravitreal aflibercept for proliferative diabetic retinopathy in the recovery study. Br. J. Ophthalmol..

[29] Fan W., Nittala M.G., Velaga S.B., Hirano T., Wykoff C.C., Ip M., Lampen S.I., van Hemert J., Fleming A., Verhoek M. (2019). Distribution of Nonperfusion and Neovascularization on Ultrawide-Field Fluorescein Angiography in Proliferative Diabetic Retinopathy (RECOVERY Study): Report 1. Am. J. Ophthalmol..

[30] Lange J., Hadziahmetovic M., Zhang J., Li W. (2018). Region-specific ischemia, neovascularization and macular oedema in treatment-naïve proliferative diabetic retinopathy. Clin. Exp. Ophthalmol..

[31] Xue K., Yang E., Chong N.V. (2016). Classification of diabetic macular oedema using ultra-widefield angiography and implications for response to anti-VEGF therapy. Br. J. Ophthalmol..

[32] Allingham M.J., Mukherjee D., Lally E.B., Rabbani H., Mettu P.S., Cousins S.W., Farsiu S. (2017). A Quantitative Approach to Predict Differential Effects of Anti-VEGF Treatment on Diffuse and Focal Leakage in Patients with Diabetic Macular Edema: A Pilot Study. Transl. Vis. Sci. Technol..

[33] Chandra S., Sheth J., Anantharaman G., Gopalakrishnan M. (2018). Ranibizumab-induced retinal reperfusion and regression of neovascularization in diabetic retinopathy: An angiographic illustration. Am. J. Ophthalmol. Case Rep..

[34] Gupta M.P., Kiss S., Chan R.V.P. (2018). Reversal of Retinal Vascular Leakage and Arrest of Progressive Retinal Nonperfusion With Monthly Anti–Vascular Endothelial Growth Factor Therapy for Proliferative Diabetic Retinopathy. Retina.

[35] Levin A.M., Rusu I., Orlin A., Gupta M.P., Coombs P., D’Amico D.J., Kiss S. (2017). Retinal reperfusion in diabetic retinopathy following treatment with anti-VEGF intravitreal injections. Clin. Ophthalmol..

[36] Leicht S.F., Kernt M., Neubauer A., Wolf A., Oliveira C.M., Ulbig M., Haritoglou C. (2014). Microaneurysm Turnover in Diabetic Retinopathy Assessed by Automated RetmarkerDR Image Analysis—Potential Role as Biomarker of Response to Ranibizumab Treatment. Ophthalmologica.

[37] Ip M.S., Zhang J., Ehrlich J.S. (2017). The Clinical Importance of Changes in Diabetic Retinopathy Severity Score. Ophthalmology.

[38] Dhoot D.S., Baker K., Saroj N., Vitti R., Berliner A.J., Metzig C., Thompson D., Singh R.P. (2018). Baseline Factors Affecting Changes in Diabetic Retinopathy Severity Scale Score After Intravitreal Aflibercept or Laser for Diabetic Macular Edema. Ophthalmology.

[39] Yu H.J., Ehlers J.P., Sevgi D.D., Hach J., O’Connell M., Reese J.L., Srivastava S.K., Wykoff C.C. (2021). Real-Time Photographic- and Fluorescein Angiographic-Guided Management of Diabetic Retinopathy: Randomized PRIME Trial Outcomes. Am. J. Ophthalmol..

[40] Wykoff C.C. (2020). PANORAMA: A Phase 3, Double-Masked, Randomized Study of the Efficacy and Safety of Aflibercept in Patients with Moderately Severe to Severe NPDR.

[41] (2020). Study of the Efficacy and Safety of Intravitreal (IVT) Aflibercept for the Improvement of Moderately Severe to Severe Non-Proliferative Diabetic Retinopathy (NPDR) (PANORAMA). https://clinicaltrials.gov/ct2/show/NCT02718326.

[42] Fahrmann E.R., Adkins L., Driscoll H.K. (2021). Modification of the Association Between Severe Hypoglycemia and Ischemic Heart Disease by Surrogates of Vascular Damage Severity in Type 1 Diabetes During 30 Years of Follow-up in the DCCT/EDIC Study. Diabetes Care.

[43] Kloecker D.E., Khunti K., Davies M.J., Pitocco D., Zaccardi F. (2021). Microvascular Disease and Risk of Cardiovascular Events and Death from Intensive Treatment in Type 2 Diabetes. Mayo Clin. Proc..

[44] Kramer C.K., Rodrigues T.C., Canani L.H., Gross J.L., Azevedo M.J. (2011). Diabetic Retinopathy Predicts All-Cause Mortality and Cardiovascular Events in Both Type 1 and 2 Diabetes: Meta-analysis of observational studies. Diabetes Care.

[45] Zhu X.-R., Zhang Y.-P., Bai L., Zhang X.-L., Zhou J.-B., Yang J.-K. (2017). Prediction of risk of diabetic retinopathy for all-cause mortality, stroke and heart failure. Medicine.

[46] Guo V.Y., Cao B., Wu X., Lee J.J.W., Zee B.C.-Y. (2016). Prospective Association between Diabetic Retinopathy and Cardiovascular Disease—A Systematic Review and Meta-analysis of Cohort Studies. J. Stroke Cerebrovasc. Dis..

[47] Sadda S.R., Nittala M.G., Taweebanjongsin W., Verma A., Velaga S.B., Alagorie A.R., Sears C.M., Silva P.S., Aiello L.P. (2020). Quantitative Assessment of the Severity of Diabetic Retinopathy. Am. J. Ophthalmol..

